# Toxicokinetic-Oriented Assessment of Nepetalactone Using In Silico ADMET Modeling, In Vitro Rat and Human Liver Microsomes, and UHPLC–MS/MS Metabolite Characterization

**DOI:** 10.3390/toxics14040319

**Published:** 2026-04-12

**Authors:** Nicolae-Bodgan Stoica, Antonio Cascajosa-Lira, Adriana Morea, Giorgiana M. Catunescu, Ruth Hornedo-Ortega, Remedios Guzmán-Guillén

**Affiliations:** 1 Faculty of Agriculture, University of Agricultural Science and Veterinary Medicine Cluj-Napoca, 400372 Cluj-Napoca, Romania; nicolae-bogdan.stoica@student.usamvcluj.ro (N.-B.S.); adriana.morea@usamvcluj.ro (A.M.); giorgiana.catunescu@usamvcluj.ro (G.M.C.); 2 Área de Toxicología, Facultad de Farmacia, Universidad de Sevilla, 41012 Sevilla, Spain; rguzman1@us.es; 3 Food Quality & Design Group, Wageningen University & Research, Bornse Weilanden 9, 6708 WG Wageningen, The Netherlands; 4 Área de Nutrición y Bromatología, Facultad de Farmacia, Universidad de Sevilla, 41012 Sevilla, Spain; rhornedo@us.es

**Keywords:** nepetalactone, toxicokinetic, metabolism, predictive toxicology, UHPLC-MS/MS

## Abstract

Nepetalactone (NL) is a volatile iridoid monoterpene widely used in biopesticidal and repellent applications, yet its toxicokinetic behavior and metabolic fate as a pure compound remain poorly characterized. This study aimed to provide an integrated toxicokinetic evaluation of NL by combining in silico absorption, distribution, metabolism, excretion and toxicity (ADMET) modeling with in vitro metabolism assays using rat and human liver microsomes, supported by UHPLC–MS/MS analysis for metabolite identification. The in silico biotransformation predicted extensive phase I oxidation followed by phase II conjugation, while ADMET predictions indicated low systemic persistence and limited toxicological concern for most metabolites. The performed in vitro microsomal assays confirmed the in silico prediction by a rapid and time-dependent NL metabolism via both oxidative (86% reduction in NL concentration after 120 min) and conjugative (89% reduction in NL concentration after 120 min) pathways in rat and human systems, with comparable depletion kinetics between species. UHPLC–MS/MS enabled the identification of multiple phase I and phase II metabolites, pointing to pronounced interspecies differences in conjugative metabolism. In this sense, while oxidoreduction and hydrolysis reactions were consistent with previously reported iridoid metabolism. This study suggests the possible formation of previously unreported amino acid-related derivatives, although these require further confirmation. Overall, these findings advance the understanding of NL biotransformation, propose a new, previously unknown, metabolic pathway for iridoids, and provide relevant data to support human health and environmental risk assessment frameworks.

## 1. Introduction

*Nepeta* essential oils (EOs) are increasingly explored as botanical repellents across public-health and integrated pest management (IPM) contexts, with demonstrated efficacy against a wide range of pests [1,2,3]. The composition of Nepeta EOs is highly polymorphic across the ~300 species described to date, giving rise to distinct chemotypes, most commonly classified as nepetalactone-rich or non-nepetalactone types (e.g., 1,8-cineole- or citronellol/geraniol-dominated profiles) [4,5]. In nepetalactone-rich chemotypes, the EO is dominated by nepetalactones (NLs) (C_10_H_14_O_2_), a group of volatile cyclopentanoid iridoid monoterpene lactones that contribute to many of the genus’ best-known bioactive properties (i.e., feline attractant activity, insect repellency) [5,6].

This chemical diversity has translated into a widening application space for NL-containing materials, spanning consumer repellents, public-health vector control, and agricultural/veterinary pest management [1,2,7,8,9]. NL-rich EOs and formulations have shown broad arthropod repellency across many species such as mosquitoes, ticks/poultry mites, bed bugs, and filth flies in laboratory assays [1,2,3,8,9,10]. In agricultural semiochemical contexts, NL was shown to be a key aphid sex pheromone component used in plant-derived lure strategies that can attract aphids and natural enemies [7]. Additionally, high NL doses were reported to repel asexual *Myzus persicae* morphs [11].

The use scenarios for NL-containing products translate into distinct human exposure routes: dermal exposure is primary for topical repellent applications, while the volatility of these oils also makes inhalation of vapors plausible during and after application [1,2,8,12]. In addition, incidental oral exposure (e.g., hand-to-mouth transfer) is a realistic pathway in non-dietary exposure assessment [13]. As NL products have also been used in pest-management The broader occupational exposure is also relevant when considering handling in various agricultural scenarios [7,11].

Toxicological information on NL-rich EOs seems to be limited. Available evidence indicates that NL-rich EOs generally exhibit low acute systemic toxicity in animal models, with high oral LD_50_ values and good dermal and inhalation tolerability [1], although mild to moderate skin and transient eye irritation have been reported depending on test conditions [1,5]. Consistent with these findings, the U.S. Environmental Protection Agency (EPA) classified refined/hydrogenated catmint (*Nepeta cataria*) EO in low acute-toxicity categories, reporting no dermal sensitization and only mild, reversible eye irritation, with subchronic nasal effects observed only at the highest oral dose tested [12]. Thus, dedicated toxicological data for pure NL are, to the best of our knowledge, absent, highlighting the need for focused evaluation of the isolated compound.

Given the limited toxicological dataset for NL-rich EOs and the lack of specific toxicological data for pure NL, expanding NL use as a biopesticide and/or repellent requires clear evidence on toxicokinetics and relevant metabolites. Accordingly, EU pesticide data require active-substance dossiers to include toxicokinetic information and consideration of relevant metabolites, and, where applicable, stereoisomer composition and potential interconversion, within the risk-assessment framework [14,15,16]. In EFSA-style frameworks, metabolism data feed directly into hazard interpretation and exposure metrics, including decisions around which residues/metabolites warrant further consideration [16]. Dermal exposure is particularly central for repellent applications, and EFSA guidance provides tiered approaches and default assumptions for dermal absorption values where chemical- and formulation-specific data are absent [17]. For plant protection products (PPP) more broadly, EFSA guidance also standardizes approaches to estimating exposure for operators/workers/residents/bystanders across scenarios, supporting harmonized non-dietary systemic exposure assessment [13].

From a test-guideline perspective, OECD TG 417 frames toxicokinetics as a core tool to characterize absorption, distribution, metabolism, excretion and toxicity (ADMET), to identify circulating moieties, and to link internal dose to observed toxicity—particularly relevant where metabolites may contribute to hazard or differ across species [14]. Understanding metabolic clearance and metabolite profiles is needed for interspecies extrapolation and for determining the specific conditions that define “relevant exposure” for non-target organisms and humans [14,16,17]. In line with the 3Rs (replacement, reduction, refinement), in vivo testing should be preceded by in silico and in vitro evidence that clarifies likely ADME pathways and key uncertainties, so that any subsequent animal work is targeted, uses fewer animals, and employs refined designs (OECD, 2010) [14]. Computational ADMET tools enable in silico prediction of metabolism and potential toxicity for NL and its metabolites, providing molecular-level insights into interactions with metabolic enzymes and biological targets [18,19]. Complementarily, in vitro liver microsomes combined with analysis by Ultra-High Performance Liquid Chromatography-Tandem Mass Spectrometry (UHPLC-MS/MS) constitute a well-established first-pass approach to assess metabolic stability, intrinsic clearance, and metabolite profiles, allowing efficient identification and prioritization of metabolites of potential toxicological relevance [20,21,22].

Taken together, the available evidence shows a notable data gap relevant to the risk assessment of PPPs: the lack of hepatic metabolic stability and metabolite identity for pure NL, particularly in human-relevant systems and in a format useful for risk assessment purposes and cross-species interpretation [1,5,12]. Accordingly, the aim of this study is to integrate for the first time in silico ADMET predictions with in vitro microsomal metabolism assays for pure NL to generate an internally consistent ADME and metabolite profile that can support human-health risk assessment for a prospective NL-based biopesticide/repellent in future public-health and IPM use scenarios. Liver microsomes from rats, as well as from humans, were employed to explore possible metabolic variations related to species. This approach sought to clarify the metabolic behavior of NL and to characterize the metabolites that may be formed. In addition, an appropriate analytical method to determine NL and identify the metabolites as part of the risk and safety assessment by UHPLC-MS/MS was validated.

## 2. Materials and Methods

### 2.1. Reagents and Chemicals

*Cis*-*trans* Nepetalactone (purity >95%; CAS: 21651-62-7) was purchased from Toronto Research Chemicals (Vaughan, ON, Canada). The following reagents were sourced from Sigma-Aldrich (St. Louis, MO, USA): phosphate-buffered saline (PBS), testosterone, 7-hydroxycoumarin, glucose-6-phosphate dehydrogenase (G6PDH), magnesium chloride (MgCl_2_), Tris buffer, and uridine 5′-diphosphoglucuronic acid (UDPGA). Methanol (MeOH) and acetonitrile (ACN) of HPLC grade were supplied by Merck (Darmstadt, Germany). Microsomal preparations included pooled rat liver microsomes from female animals and human liver microsomes, obtained from Corning Gentest (Woburn, MA, USA) and Gibco (Biomol, Sevilla, Spain), respectively. Alamethicin, NADP^+^, and glucose-6-phosphate (G6P) were acquired from Cayman Chemical Company (Ann Arbor, MI, USA). All solutions were prepared using ultrapure water (18.2 MΩ·cm) generated by a NANOpure Diamond™ purification system (Barnstead, NH, USA).

### 2.2. In Silico Biotransformation of NL

BioTransformer 4.0, a freely available web-based software for small-molecule metabolism prediction developed by the Wishart Research Group (University of Alberta, Canada), was used to predict potential metabolites. The molecular structure of NL, provided in canonical SMILES format, was submitted to the software to generate its metabolomic profile. The prediction parameters included setting the “Number of Reaction Interactions to Calculate” to 3 and selecting the “EC-based transformation” option, following previously reported methodologies [19].

### 2.3. Toxicity Prediction of NL and Its Metabolites

Following metabolite structure prediction, the canonical SMILES of NL and its predicted metabolites were submitted to ADMETlab 3.0 to evaluate a broad range of physicochemical, pharmacokinetic, and toxicological parameters. The predicted kinetic endpoints included Caco-2 and MDCK permeability, human intestinal absorption (HIA), plasma protein binding (PPB), plasma clearance (CLplasma), half-life (T_1/2_), and blood–brain barrier (BBB) penetration. In addition, ADMETlab 3.0 was used to estimate multiple toxicity-related endpoints, including carcinogenicity, human hepatotoxicity, nephrotoxicity, neurotoxicity, genotoxicity, AMES mutagenicity, skin sensitization, eye irritation, respiratory toxicity, and selected environmental toxicity parameters (IG50) for *Tetrahymena pyriformis,* LC50 for fathead minnow and for *Daphnia magna*, and bioconcentration factors. The software was also employed to assess the potential of NL and its predicted metabolites to act as inhibitors or substrates of the major human cytochrome (CYP) P450 isoforms CYP1A2, CYP2C19, CYP2C9, CYP2D6, and CYP3A4, with the associated probabilities expressed as percentages [23].

### 2.4. Analytical Method Validation

The UHPLC–MS/MS method for the determination of NL was initially validated in solvent to assess the instrumental performance in terms of linearity, sensitivity, precision, and recoveries, in accordance with established analytical recommendations by AOAC (2016) and EURACHEM (2016) [24,25,26]. For this purpose, a calibration curve was performed using *cis*-*trans* NL dissolved in 50% ACN, over a concentration interval of 100–1000 µg L^−1^. Detection and quantification limits (LOD and LOQ) values were estimated from the residual standard deviation of the regression curve obtained from the calibration curve, applying the expressions 3 × SD/slope and 10 × SD/slope, respectively, as described in Miller and Miller (2000) [27] and the ICH guideline [28]. Recovery and precision studies were carried out in triplicate at each concentration level (100, 500, and 1000 µg L^−1^) over three separate days, covering the entire calibration range. For each concentration level, a one-way analysis of variance (ANOVA) was applied to estimate within-day (S_W_) and between-day (S_B_) repeatability and to calculate the intermediate precision (S_IP_) and its relative standard deviation (%RSD_IP_) and later compared with the acceptance criteria established by AOAC (2016) [24] for each concentration level. These values reflect the instrumental sensitivity of the method in solvent, and no biological matrix was involved at this stage.

### 2.5. Rat and Human Microsomes Metabolism of NL

The in vitro hepatic metabolism of NL was investigated using an experimental design adapted from previously published protocols [19,29,30,31]. Female rat liver microsomes (FRLM) and human liver microsomes (HLM) were selected to assess species-specific metabolic behavior. All incubations were conducted in a final reaction volume of 1 mL, consisting of microsomal protein suspended in 100 mM phosphate-buffered saline (PBS) at pH 7.4.

To initiate phase I biotransformation, microsomal suspensions were equilibrated at 37 °C prior to the addition of NL (final concentration: 5 µM; 831 ppb). Metabolic reactions were triggered by introducing an NADPH-regenerating system containing NADP^+^, glucose-6-phosphate, glucose-6-phosphate dehydrogenase, and MgCl_2_. Phase II metabolism was subsequently explored by supplementing the reaction mixtures with UDP–glucuronic acid (UDPGA) under optimized glucuronidation conditions, including UDP–glucuronosyltransferase, Tris–HCl buffer (pH 7.5), MgCl_2_, alamethicin, and UDPGA.

Incubations were carried out at 37 °C, and aliquots were collected at 30, 60, and 120 min to monitor metabolic progression over time. Enzyme functionality was confirmed using testosterone (20 µM) as a reference substrate for oxidative metabolism and 7-hydroxycoumarin (1 µM) as a marker compound for glucuronidation. Metabolic reactions in the taken aliquots (100 µL) were terminated by the addition of an equal volume of ice-cold ACN (hence, diluted 1/2), and samples were preserved for subsequent UHPLC–MS/MS analysis. A blank without NL was also included in each reaction (*n* = 3, per reaction).

For quantification of NL in microsomal incubation samples, a matrix-matched calibration approach was employed. Calibration curves were prepared in microsomal systems independently for each reaction mixture (for oxidation or glucuronidation) to account for potential matrix effects (ME). These were evaluated by comparing the calibration curves prepared in solvent and in matrix for both oxidation and glucuronidation, according to Equation (1).

(1) $$ME\;(\%)=100\times (\frac{{\mathrm{slope}}_{\mathrm{matrix}}}{{\mathrm{slope}}_{\mathrm{solvent}}}-1)$$

### 2.6. UHPLC-MS/MS Analysis

Chromatographic separation was carried out using an UltiMate 3000 binary UHPLC system (Thermo Fisher Scientific) (Waltham, Massachusetts, USA) coupled to a Q Exactive high-resolution Orbitrap mass spectrometer (Thermo Fisher Scientific) equipped with a heated electrospray ionization (HESI) source operating in positive ionization mode. The separation was performed on an Acquity BEH C18 reversed-phase column (100 × 2.1 mm, 1.7 μm particle size), maintained at 35 °C. The injection volume was 5 μL and the mobile phase flow rate was set at 0.4 mL min^−1^. The mobile phase consisted of solvent A (water containing 0.1% formic acid) and solvent B (methanol containing 0.1% formic acid). The following gradient elution program was applied: initial conditions of 5% B were maintained from 0.0 to 0.5 min; solvent B was then linearly increased to 100% from 0.5 to 5.0 min; 100% B was held until 7.0 min, followed by re-equilibration to 5% B at 7.1 min, which was maintained until the end of the run at 10.0 min.

Mass spectrometric detection was performed using a full MS/data-dependent MS^2^ (dd-MS^2^, TopN = 5) acquisition method. Full-scan mass spectra were acquired over an *m*/*z* range of 60–900 at a resolution of 70,000 (FWHM), with an AGC target of 3 × 10^6^ and a maximum injection time of 100 ms. Data-dependent MS^2^ spectra were acquired at a resolution of 17,500, using an isolation window of 0.7 *m*/*z*, an AGC target of 2 × 10^5^, and a maximum injection time of 50 ms. Stepped normalized collision energies (NCE) of 30, 40, and 60 were applied. The electrospray ionization source was operated under the following optimized conditions: spray voltage, 3.5 kV; capillary temperature, 320 °C; probe heater temperature, 425 °C; sheath, auxiliary and sweep gas flow, 50, 13 and 3 respectively (arbitrary units); and S-Lens RF level set to 50. Dynamic exclusion was enabled with an exclusion time of 6 s to improve MS^2^ spectral coverage.

### 2.7. Metabolite Identification

Data processing for phase I and phase II metabolism studies was carried out using Compound Discoverer™ version 3.2 (Thermo Fisher Scientific, Waltham, MA, USA). Raw UHPLC–MS/MS data were imported into the software for feature detection, spectral filtering, and retention time alignment across samples. As authentic standards of NL metabolites were not available, metabolite annotation was performed by comparing the exact mass of the parent compound with the theoretical masses generated from multiple possible phase I and phase II biotransformation reactions. These transformations were automatically proposed by the software and supported by high-resolution mass accuracy and diagnostic fragmentation patterns. Additional confidence in metabolite assignment was obtained through database searches in MassBank, mzCloud, ChemSpider, and an in-house laboratory database. A mass tolerance of ±5 ppm was applied for all compound annotations. The identification of metabolites should be regarded as tentative, owing to the unavailability of authentic reference standards and the absence of essential cofactors required to support certain phase II metabolic reactions.

### 2.8. Statistics

Data from the in vitro experiments are expressed as mean values of 3 repetitions accompanied by their standard deviation (SD). Statistical analyses were performed using GraphPad Prism 9 (GraphPad Software Inc., La Jolla, CA, USA). The distribution of the data was first examined using the Kolmogorov–Smirnov test. When normality was confirmed, comparisons were carried out by one-way ANOVA followed by Tukey–Kramer post hoc testing where appropriate. When the data did not follow a normal distribution, the Kruskal–Wallis test was applied, and pairwise differences were explored using Dunn’s test. Statistical significance was established at *p* < 0.05.

## 3. Results and Discussion

### 3.1. Predicted Biotransformation Pathway

**Figure 1:**
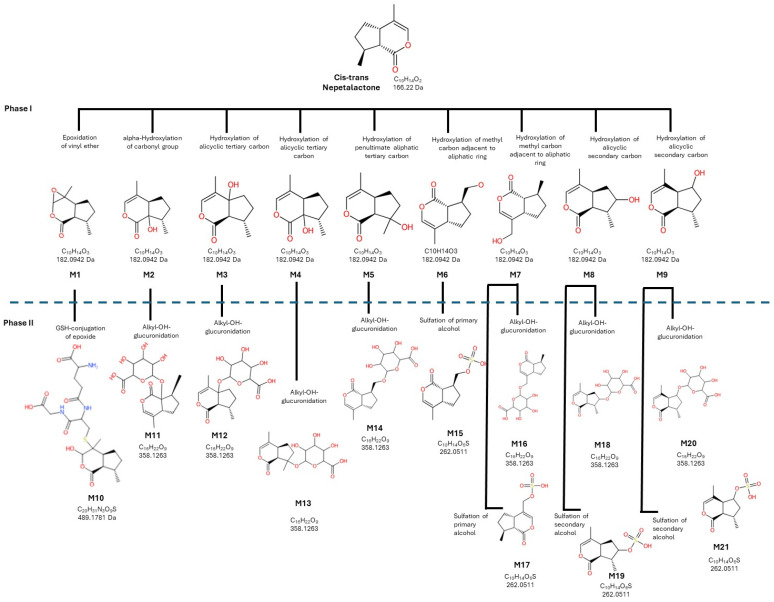
Predicted metabolic pathway of Nepetalactone according to the in silico metabolism assay performed with Biotransformer 4.0 software.

The first aim of this study was to characterize the potential phase I and phase II metabolic pathways of NL using Biotransformer 4.0. Figure 1 illustrates the proposed predicted phase I and phase II metabolic profile of NL. A total of 21 metabolites (M1–M21) were tentatively identified and classified according to their in silico biotransformation reactions.

Phase I metabolism of NL predominantly involved oxidative reactions, generating/producing 9 primary metabolites (M1–M9). These transformations included epoxidation of the vinyl ether moiety (M1), α-hydroxylation of the carbonyl group (M2), hydroxylation at alicyclic tertiary carbons (M3 and M4), hydroxylation at the penultimate aliphatic tertiary carbon (M5), and hydroxylation of methyl or methylene groups adjacent to the aliphatic ring (M6–M9). All phase I metabolites exhibited an increase of +16 Da relative to the parent compound, consistent with mono-oxygenation reactions.

Phase II metabolism mainly consisted of conjugation reactions involving glutathione (GSH), glucuronic acid, and sulfate. One GSH conjugate (M10) was detected, corresponding to the conjugation of GSH to an epoxide intermediate. Multiple glucuronide conjugates (M11–M14, M16, M18, and M20) were observed, derived from hydroxylated phase I metabolites through alkyl-OH glucuronidation. Sulfation reactions were also identified, generating sulfate conjugates of primary and secondary alcohols (M15, M17, M19, and M21). These phase II metabolites showed the expected mass increases associated with glucuronidation (+176 Da) and sulfation (+80 Da), respectively.

Therefore, the potential metabolic profile of NL was predicted by extensive oxidative phase I transformations followed by conjugation reactions in phase II, indicating multiple metabolic hotspots across the molecule.

**Figure 2:**
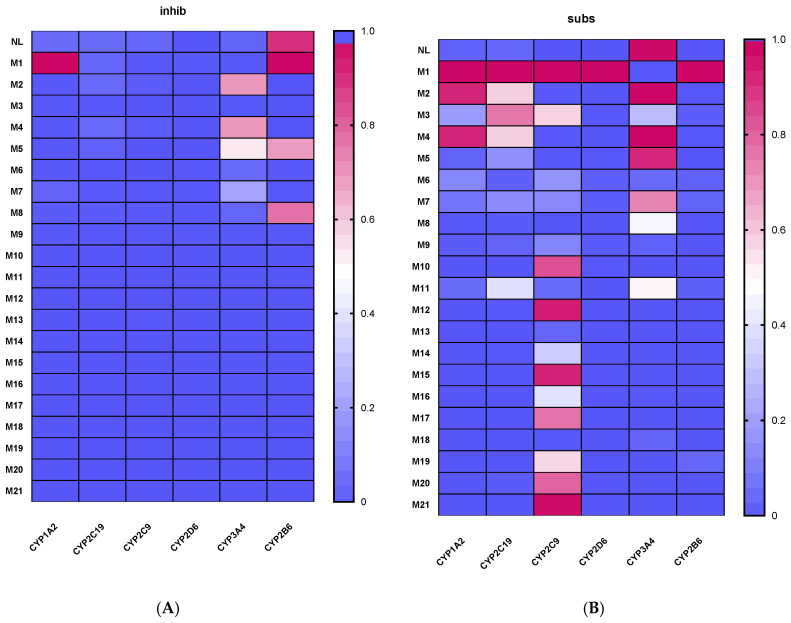
Heatmap of the predicted probability of Nepetalactone and its metabolites (M1–M21) acting as inhibitors (**A**) or substrates (**B**) of different CYP450 isoforms.

Figure 2 shows the predicted probabilities of NL and its phase I and phase II metabolites (M1–M21) to act as substrates or inhibitors of major human CYP450 isoforms, as estimated by using ADMETlab 3.0. The results are presented as heatmaps, where color intensity reflects the relative probability associated with each CYP–compound interaction.

Overall, NL exhibited a low probability of interaction with selected CYP isoforms, either as a substrate or inhibitor. The only cases with a high probability of interaction were the CYP2B6 as an inhibitor and CYP3A4 as a substrate. In contrast, several phase I metabolites displayed increased predicted probabilities for CYP substrate interaction, particularly for specific isoforms (CYP1A2, CYP2C19 and CYP 3A4), suggesting that oxidative biotransformation may enhance the likelihood of enzyme binding. The predicted interaction profiles varied markedly among individual metabolites, indicating that the position and nature of hydroxylation strongly influence CYP isoform recognition.

Phase II metabolites, including glucuronide, sulfate, and GSH conjugates, generally showed lower predicted probabilities of acting as CYP substrates or inhibitors compared to phase I metabolites. This tendency was consistent across most CYP isoforms, reflecting the reduced affinity of highly polar conjugated metabolites for CYP-mediated metabolism. But in the case of CYP2C9, there is an increase in the probability of interactions, especially for sulfated metabolites.

These results highlight substantial differences in CYP interaction potential between NL and its metabolites, with phase I transformations increasing and phase II conjugations generally decreasing the predicted likelihood of CYP involvement.

The appearance of several positional +16 Da isomers is characteristic of CYP-mediated Phase I oxidation and is a common outcome in metabolite identification workflows [21]. Although earlier NL-rich EO toxicology studies did not identify metabolites, their low acute systemic toxicity and lack of bioaccumulation concern shown by Zhu et al. [1] and the U.S. EPA [12] are most compatible with efficient oxidative metabolism that increases molecular hydrosolubility and promotes rapid excretion rather than metabolic stability. In line with this interpretation, the Phase II profile glucuronidation (+176 Da) of hydroxylated metabolites, sulfation (+80 Da) of alcohols, and GSH trapping consistent with epoxide detoxification matches the conjugation pathways. This aligns with the OECD TG 417 guideline and the data requirements set out in Commission Regulation (EU) No 283/2013 for detoxification-oriented clearance of oxidized plant secondary metabolites [14,15,16,21,30].

The present in silico screening suggested a low probability that NL and its phase I/II metabolites would interact with major human CYP isoforms. While NL-specific CYP interaction data are, to the best of our knowledge, absent from the *Nepeta* toxicity literature, this prediction is consistent with the mixture-based safety phenotype and regulatory evaluations of catmint, which did not raise systemic interaction concerns. These findings align with EU/OECD ADME frameworks that consider enzyme induction/inhibition as a potential but case-dependent toxicokinetic issue [1,12,14,15].

This metabolic profile also aligns with general metabolism observations for other iridoids, such as genipin (found in the fruits of *Gardenia jasminoides*), where conversion to more polar conjugates (e.g., sulfates or glucuronides) predominates over persistence of the unmetabolized parent compound [32]. Comparable Phase II outcomes have been reported for other iridoids in human-relevant in vitro systems: verproside (found in *Veronica officinalis* and other *Veronica* or *Pseudolysimachion* species) formed multiple glucuronide and sulfate conjugates in human hepatocytes, reinforcing conjugation as a major clearance route for iridoid scaffolds [33].

### 3.2. In Silico Predicted Toxicity

**Figure 3:**
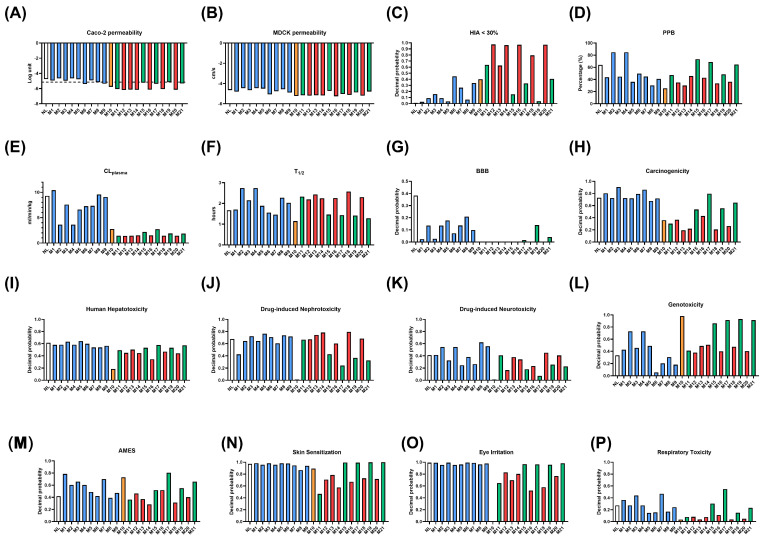
Predictions of different kinetic and toxicological parameters of Nepetalactone and its metabolites (M1–M21). (**A**) Caco-2 permeability (Log units); optimal: higher than −5.15 Log unit. (**B**) MDCK permeability (cm/s); low permeability: <2 × 10^−6^ cm/s; medium permeability: 2–20 × 10^−6^ cm/s; high passive permeability: >20 × 10^−6^ cm/s. (**C**) Human Intestinal Absorption, within the range of 0 to 1, showing the probability of being absorbed; (**D**) Plasma Protein Binding (%); optimal: <90%; (**E**) Plasma clearance (CL plasma; mL/min/kg); high clearance: >15 mL/min/kg; moderate clearance: 5–15 mL/min/kg; low clearance: <5 mL/min/kg; (**F**) Half-life (T1/2, hours); ultra-short half-life: <1 h; short half-life: 1–4 h; intermediate short half-life: 4–8 h; long half-life: >8 h; (**G**) Blood–Brain Barrier (BBB) penetration, within the range of 0 to 1, where 0: BBB (−), and 1: BBB (+); (**H**) Carcinogenicity, within the range of 0 to 1, where 0: non-carcinogens, and 1: carcinogens; (**I**) Human Hepatotoxicity, within the range of 0 to 1, where 0: non-hepatotoxic, and 1: hepatotoxic; (**J**) Drug-induced Nephrotoxicity, within the range of 0 to 1, where 0: non-nephrotoxic, and 1: nephrotoxic; (**K**) Drug-induced Neurotoxicity, within the range of 0 to 1, where 0: non-neurotoxic, and 1: neurotoxic; (**L**) Genotoxicity, within the range of 0 to 1, where 0: non-genotoxic, and 1: genotoxic; (**M**) AMES test, within the range of 0 to 1, where 0: AMES (−), and 1: AMES (+); (**N**) Skin sensitization, within the range of 0 to 1, where 0: non-sensitizer, and 1: sensitizer; (**O**) Eye irritation, within the range of 0 to 1, where 0: non-irritant, and 1: irritant; (**P**) Respiratory toxicant, within the range of 0 to 1, where 0: respiratory toxicant, and 1: respiratory toxicant. Note: Colors indicate metabolite classes: Unmetabolized Nepetalactone (in white), phase I oxidized metabolites (in blue), phase II GSH-conjugates (in yellow), phase II sulfate conjugates (in green), phase II glucuronic acid conjugates (in red).

To further support the comprehensive evaluation of NL, the kinetic behavior and toxicological properties of the parent compound and its predicted metabolites were assessed. Figure 3 provides a detailed overview of the predicted kinetic and toxicological profiles of NL and its metabolites, grouped according to their metabolic origin: phase I oxidized metabolites (blue), sulfate conjugates (green), GSH conjugates (yellow), and glucuronide conjugates (red).

Regarding kinetic parameters, phase I metabolites (in blue) generally exhibited physicochemical and absorption-related profiles comparable to or moderately altered relative to NL. In several cases, oxidized metabolites showed similar or slightly reduced predicted Caco-2 (Figure 3A) and MDCK permeability (Figure 3B). In contrast, the probabilities of HIA were notably increased by 10–50%, reflecting the increase in polarity introduced by CYP450 reactions. On the other hand, phase II conjugation reactions influenced these parameters. Sulfated metabolites (in green) consistently displayed higher predicted membrane permeability and intestinal absorption (Figure 3C), indicating the impact of sulfate conjugation on transport-related properties. A similar but more pronounced effect was observed for glucuronide conjugates (in red), which exhibited the highest predicted permeability values across most endpoints of absorption. The GSH conjugate (in yellow) also showed an increased permeability and absorption compared to NL and phase I metabolites.

The parameters related to systemic disposition and excretion, including PPB (Figure 3D), plasma clearance (CLplasma) (Figure 3E), and half-life (Figure 3F), were also influenced by the type of metabolic transformation. Phase I metabolites displayed heterogeneous profiles, with some oxidized derivatives showing predicted plasma clearance and half-life values comparable to NL, while others such as M1–M4 deviated depending on the position of hydroxylation. In contrast, sulfate and glucuronide conjugates generally exhibited shorter predicted plasma clearance (∼0.1 mL/min/kg) and higher half-lives (∼2 h), suggesting reduced systemic persistence. The GSH conjugate followed a similar tendency, showing elevated clearance and limited predicted half-life.

Predicted BBB penetration (Figure 3G) further highlighted the influence of metabolite class. NL and several phase I metabolites retained moderate predicted probabilities of BBB penetration, whereas sulfate, glucuronide, and GSH conjugates consistently showed very low predicted BBB penetration, reflecting the impact of conjugation reactions on limiting central nervous system (CNS) exposure.

Toxicological predictions revealed clear class-dependent grouping. Predicted carcinogenicity (Figure 3H) scores for NL and its metabolites remained low at 60%, suggesting a non-potential carcinogenic profile. None of the metabolites reached probability values indicative of strong carcinogenic concern (higher than 80%). Conjugated metabolites, particularly glucuronide and sulfate derivatives, consistently showed the lowest carcinogenicity predictions (under 30%). Most compounds displayed a similar predicted human hepatotoxicity (Figure 3I), with NL and oxidized metabolites showing higher values compared to conjugated forms. Predicted drug-induced nephrotoxicity (Figure 3J) was generally high for NL and its metabolites. GSH metabolite presented especially lower probabilities than others (0%). Neurotoxicity (Figure 3K) predictions were predominantly low, especially for GSH conjugate. Although some oxidized metabolites showed modest increases in probability, these remain under 0.6, associated with low neurotoxic potential, aligning with the limited predicted BBB penetration observed for most metabolites.

Both genotoxicity (Figure 3L) and AMES mutagenicity (Figure 3M) predictions indicated no significant genotoxic concern for NL. However, some probability values increased for some oxidized GSH-conjugates and sulfated metabolites, indicating a DNA-reactive metabolic profile.

Skin sensitization (Figure 3N) potential was predicted to be high overall. A small number of glucuronic metabolites showed slightly decreased probabilities (60–80%) compared with NL (∼100%); however, they still exhibited high sensitization risk. The predictions also suggested high eye irritation potential (Figure 3O) for NL and its metabolites. All compounds, except for the GSH and glucuronic conjugates, demonstrated a probability of 80–100% consistent with strong ocular irritancy, indicating a generally mild irritation profile.

Respiratory toxicity (Figure 3P) probabilities remained low across NL and its metabolic derivatives. Oxidized metabolites showed marginal increases compared to conjugates, but none reached levels indicative of respiratory hazard (>50%). This broadly agrees with the mixture-based records showing transient eye irritation, skin irritation and low inhalation/respiratory hazard for NL-rich EO [1,12]. But the skin sensitization signal found in this study seems more conservative than the U.S. EPA assessment of refined/hydrogenated catmint oil, which reported no dermal sensitization [1,12].

**Figure 4:**
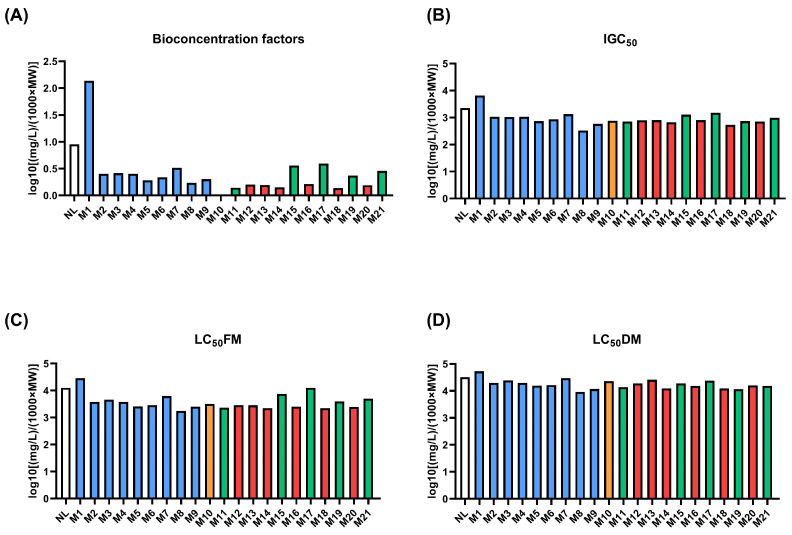
Predictions of different environmental toxicity parameters of Nepetalactone and its metabolites (M1-M21). (**A**) Bioconcentration Factors; (**B**) *Tetrahymena pyriformis* 50 percent growth inhibition concentration (IGC_50_); (**C**) 96 h fathead minnow 50 percent lethal concentration (LC_50_FM); (**D**) 48 h *Daphnia magna* 50 percent lethal concentration (LC_50_DM). Note: Colors indicate metabolite classes: Unmetabolized Nepetalactone (in white), phase I oxidized metabolites (in blue), phase II GSH-conjugates (in yellow), phase II sulfate conjugates (in green), phase II glucuronic acid conjugates (in red).

To further expand the safety assessment of NL, its potential environmental impact was evaluated, considering both the parent compound and its predicted phase I and phase II metabolites. Figure 4 illustrates the predicted environmental toxicity profiles of NL and its phase I and phase II metabolites (M1–M21), enabling a comparative evaluation of how metabolic transformations influence environmental fate and aquatic toxicity.

The predicted bioconcentration factors (BCF; Figure 4A) showed marked differences among metabolite classes. NL and especially M1 (oxidized metabolite) exhibited higher predicted BCF values, indicating a greater potential for bioaccumulation. In contrast, several phase I and all phase II conjugates, particularly glucuronides and sulfate conjugates, displayed substantially lower predicted BCF values (<0.2 log10 [(mg/L)/(1000 × MW)]). The GSH conjugate also showed reduced bioconcentration potential relative to NL (0.954 log10 [(mg/L)/(1000 × MW)]), reflecting the increased polarity and reduced lipophilicity associated with conjugation reactions.

The predictions for *Tetrahymena pyriformis* growth inhibition (IGC_50_; Figure 4B) showed that NL and its phase I metabolites generally exhibited similar IGC_50_ values, a tendency that was also reflected in aquatic organism lethality predictions. For fathead minnow acute toxicity (LC_50_FM; Figure 4C), several phase I and phase II metabolites (M2–M21) displayed slightly lower predicted LC_50_ values than NL (4.104 log10 [(mg/L)/(1000 × MW)]), indicating marginally higher predicted sensitivity (3.53 ± 0.21 log10 [(mg/L)/(1000 × MW)]). Likewise, predictions for *Daphnia magna* acute toxicity (LC_50_DM; Figure 4D) revealed toxicity profiles comparable to that of NL.

Overall, across all evaluated aquatic toxicity endpoints, NL and its metabolites demonstrated highly similar predicted toxicity profiles. To the best of our knowledge, this study represents the first report specifically addressing the ecotoxicological effects of iridoids. The consistently low ecotoxicological effects observed across trophic levels align with a low environmental persistence, which is most plausibly explained by the pronounced metabolic liability of iridoids [34].

### 3.3. UHPLC-MS/MS Method Performance

**Figure 5:**
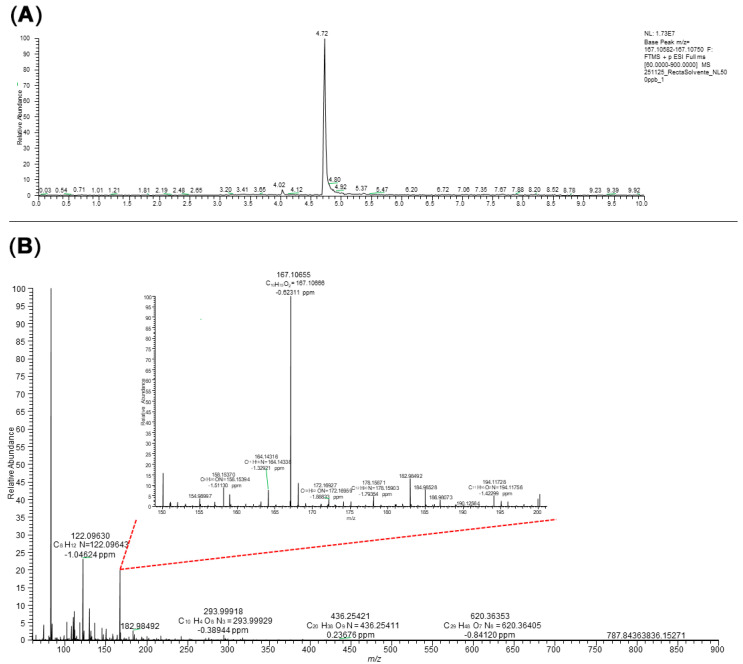
MRM chromatogram of Nepetalactone (500 μg L^−1^) by UHPLC–MS/MS (**A**), and its mass spectra (**B**) of the ion at *m*/*z* 167.10655 corresponding to the protonated molecule [M + H]^+^ (C_10_H_15_O_2_^+^).

**Figure 6:**
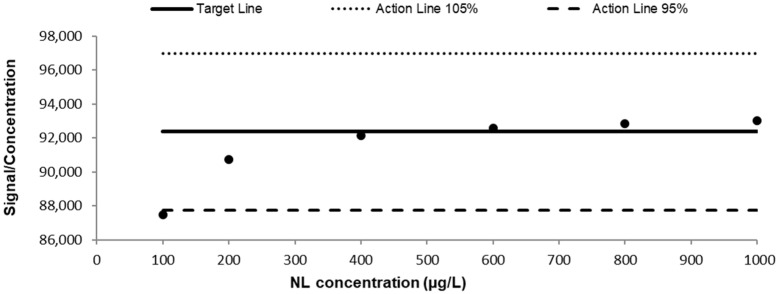
Response linearity of Nepetalactone in 50% ACN (Huber plot).

To experimentally characterize the analytical performance of NL, its chromatographic profile was evaluated under the established instrumental conditions. The chromatographic behavior of NL is shown in Figure 5. The analytical response was determined for prepared standards of NL in 50% ACN, with a 6-point calibration curve (100, 200, 400, 600, 800 and 1000 µg L^−1^), resulting in the following regression equation: y = 93,596x − 588,446 (R^2^ = 0.9997). The corresponding Huber plot was obtained with the 6-point calibration curve (Figure 6). These results indicate that the method exhibited linearity across the tested concentration ranges. The calculated recoveries (%), repeatability, intermediate precision values, LOD and LOQ for NL are presented in Table 1. It should be noted that the LOQ estimated from regression statistics may be lower than the lowest calibration standard, as it represents the theoretical quantification capability of the instrument, whereas the calibration range defines the validated working interval. All the obtained RSD values were below the acceptable range tabulated by AOAC (≤11–16% for 100 and 500 µg L^−1^, and ≤8% for 1000 µg L^−1^), and the same applies to recoveries, all being within the required range of 80–110% for the 3 concentrations assayed. Thus, the method can be considered acceptable in terms of trueness and yields. The results indicated a mild matrix effect (12.4%) under oxidative conditions and a strong matrix effect (56.7%) under glucuronidation conditions. These findings highlight the influence of the biological matrix on the analytical signal and justify the use of matrix-matched calibration for accurate quantification.

### 3.4. In Vitro Rat and Human Metabolism of NL

**Figure 7:**
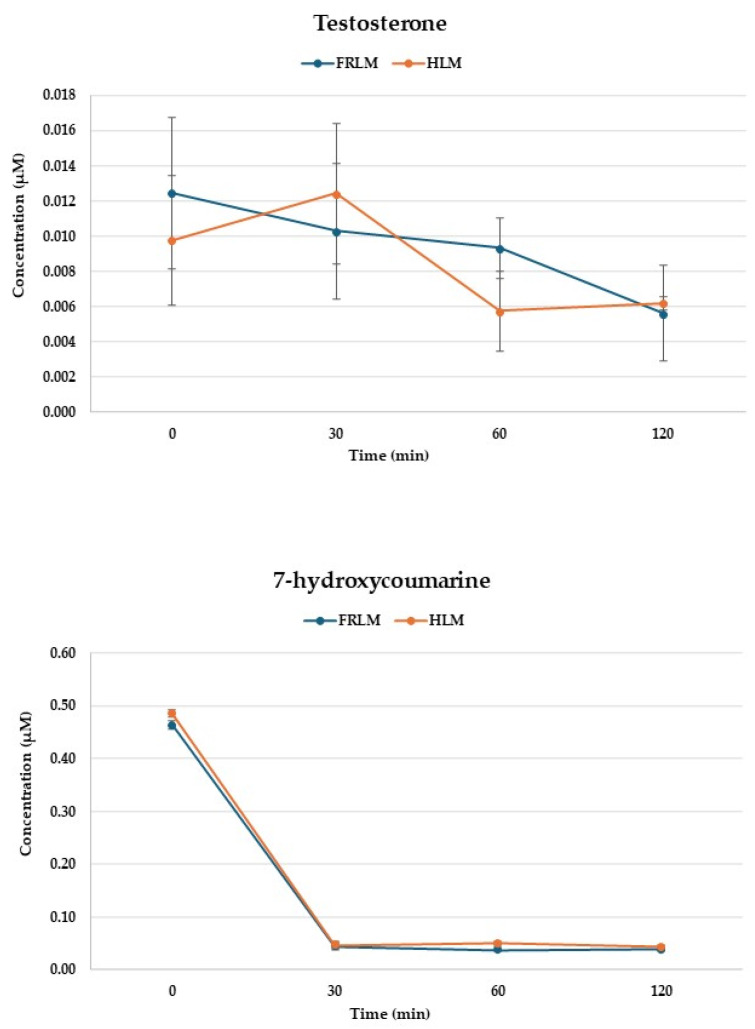
Metabolic stability of testosterone and 7-hydroxycoumarine in pooled female rat and human liver microsomes (FRLM and HLM). Data are expressed as the means ± SD of triplicate runs.

To investigate the in vitro metabolic processes under the experimental conditions employed, metabolic assays were conducted using established liver microsomal systems. Figure 7 illustrates the metabolic behavior of testosterone and 7-hydroxycoumarin, used as positive control substrates for phase I and phase II metabolism, respectively, in FRLM and HLM. Testosterone, a well-established phase I oxidative metabolism marker, showed a gradual decrease in concentration over time in both FRLM and HLM incubations, decreasing from 4 to 2 mg L^−1^. Comparable temporal profiles between rat and human microsomes confirm adequate enzymatic activity and functional consistency of the microsomal preparations. On the other hand, 7-hydroxycoumarin, a canonical phase II conjugation substrate, exhibited a rapid decline in concentration within the first 30 min in both microsomal systems, followed by low residual levels at later time points. This rapid depletion is consistent with efficient conjugative metabolism, primarily via glucuronidation [29], and validates the functionality of phase II metabolic enzymes in both FRLM and HLM.

**Figure 8:**
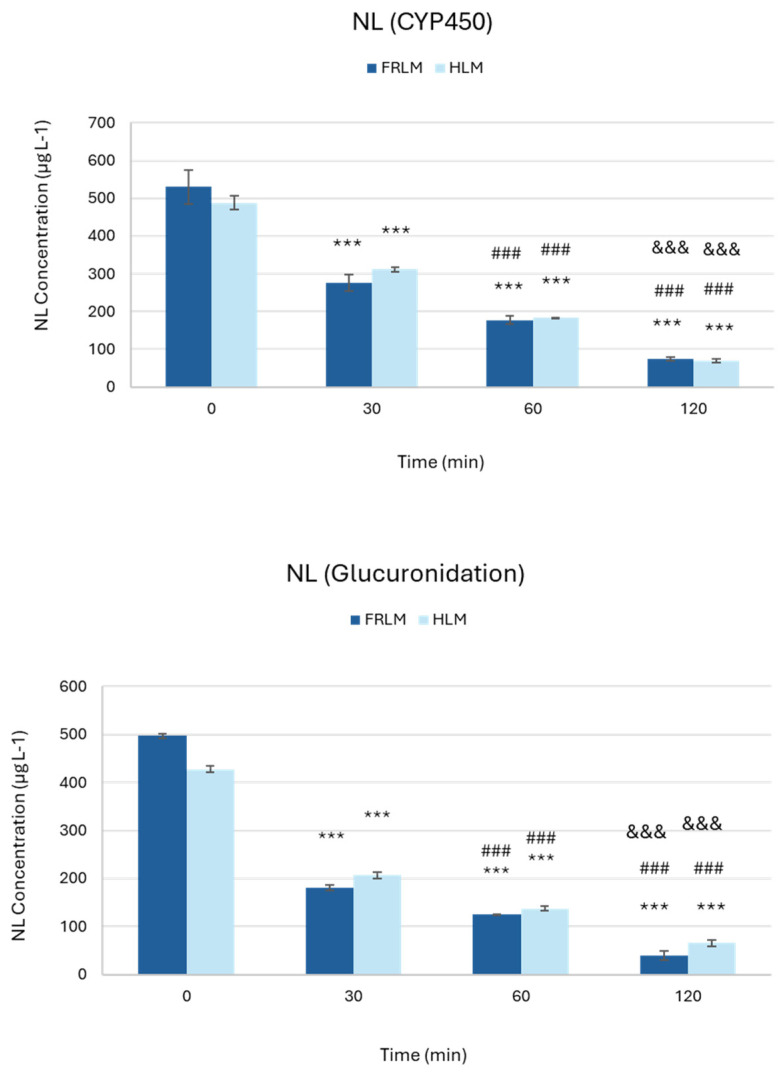
Metabolic stability of Nepetalactone incubated with female rat and human liver microsomes (FRLM and HLM) under the two metabolic reaction conditions: phase I CYP450; phase II glucuronidation. Data are expressed as the means ± SD of triplicate runs. *** statistically significant vs. 0 min; ### statistically significant vs. 30 min; and &&& statistically significant vs. 60 min (*p* < 0.0001).

Figure 8 depicts the time-dependent metabolic stability of NL following incubation with FRLM and HLM under phase I (CYP450-mediated oxidation) and phase II (glucuronidation) reaction conditions. Under phase I CYP450 conditions, NL concentration decreased progressively over time in both FRLM and HLM incubations, indicating active oxidative metabolism. A significant reduction (*p* < 0.0001) in NL levels was already evident at 30 min, with a 42% decrease compared with time zero, followed by further significant decreases at 60 min (65% decrease; *p* < 0.0001) and 120 min (86% decrease), all relative to time zero. Overall, the temporal profiles were similar between FRLM and HLM, no statistical significance was found. Similarly, under phase II glucuronidation conditions, NL exhibited a significant and time-dependent decrease in concentration in both microsomal systems. Statistically significant reductions (*p* < 0.0001) were observed as early as 30 min, with a 58% decrease compared to time zero, with continued decreases at 60 min (72% decrease) and 120 min (89% decrease), compared to time zero. No statistical differences were found between FRLM and HLM metabolism.

Therefore, these results demonstrate that NL undergoes rapid and progressive metabolism through both phase I oxidative and phase II conjugative pathways in rat and human liver microsomes, confirming its susceptibility to hepatic biotransformation and supporting the relevance of both metabolic routes.

Nepetalactone, like other naturally occurring iridoids (such as picroside or genipin), appears to exhibit rapid metabolic turnover, a feature that is consistent with multiple in vivo pharmacokinetic studies on structurally related iridoid and secoiridoid glycosides [34]. These studies consistently report fast oral absorption followed by a rapid decline in plasma concentrations, with parent iridoids and/or primary metabolites appearing transiently in circulation and being largely eliminated within approximately 2 h post-dosing [35,36,37]. Such short systemic residence times are indicative of efficient oxidative and conjugative metabolism, supporting the interpretation that NL shares the low persistence and high metabolic liability characteristic of plant-derived iridoids rather than exhibiting metabolic stability or prolonged systemic exposure.

### 3.5. In Vitro Metabolite Identification

The metabolic profiling of NL in FRLM and HLM led to the identification of several potential phase I and phase II metabolites based on accurate mass measurements, retention times, and predicted biotransformations (Table 2).

Phase I metabolism primarily involved reduction and hydration-related reactions. Two desaturated or hydrated metabolites (M22 C_10_H_12_O_2_ and M23 C_10_H_16_O_3_) were detected in both FRLM and HLM, indicating conserved oxidative pathways across species. Reduced metabolites (M24 C_10_H_16_O_2_) were observed exclusively in HLM, suggesting a human-specific reduction pathway not evident in rat microsomes.

Phase II metabolism revealed a greater diversity of conjugated metabolites, predominantly detected in HLM. Phase II conjugation is also commonly reported for other iridoids in human-relevant in vitro systems [33], supporting a detoxification-oriented clearance framework. In this context, several features were tentatively annotated as cysteine-related adducts (M25, M26, and M33) based on accurate mass shifts and fragmentation patterns. These metabolites were identified only in human microsomes, which could be consistent with thiol-related adduct formation; however, this assignment remains tentative (see discussion below). Additional features were tentatively annotated as amino acid-related adducts, including glycine (M27 and M28) and arginine-related species (M34 and M35), based on mass spectral interpretation, and were likewise exclusive to HLM, highlighting pronounced species-dependent differences in conjugative metabolism. A stearyl-conjugated metabolite (M30) was also detected solely in human microsomes and showed one of the highest signal intensities among phase II metabolites. Only one phase II metabolite (M29; C_12_H_20_O), involving reduction and acetylation, was detected in FRLM but not in HLM, suggesting a rat-specific metabolic pathway. Metabolites M25 and M33 were found to share identical accurate masses, suggesting that these compounds are structural isomers.

These results demonstrate that NL undergoes both phase I and phase II biotransformation in rat and human liver microsomes, with phase I pathways largely conserved between species, whereas phase II metabolism is more extensive and diverse in human microsomes, underscoring relevant interspecies differences in NL metabolic fate.

Importantly, the microsomal system used in this study consisted of washed liver microsomes, and no exogenous glutathione, cysteine, amino acids, or specific phase II cofactors (e.g., PAPS) were added to the incubations. Therefore, the formation of enzymatic thiol or amino acid conjugates cannot be confirmed under the experimental conditions employed. The observed features assigned as conjugates should thus be interpreted with caution and are more appropriately considered as tentative annotations, which may also arise from non-enzymatic adduct formation, matrix-related processes, or limitations inherent to high-resolution mass spectrometry-based identification without authentic reference standards.

The in silico analysis predicted the formation of several metabolites based on computational biotransformation models. However, these predicted metabolites did not correspond to those identified in the microsomal incubations. This discrepancy may be explained by the inherent limitations of in silico approaches [38], which do not account for certain phase I reactions and do not consider the possibility of amino acid conjugation. Thereby, they limit the ability of the in silico analysis to fully replicate the complexity of microsomal metabolism.

A limitation of the present study is that the assignment of metabolites M22–35 as metabolites is based solely on mass spectrometric evidence, without definitive confirmation of their origin from the parent compound. The most robust approach to unequivocally establish their metabolic provenance is the use of isotopically labeled substrates (e.g., ^13^C- or deuterium-labeled parent compound), which allows direct tracking of the biotransformation products [14].

Although oxidoreduction and hydrolysis reactions have been previously reported for other iridoids in pharmacokinetic studies [32,34], this is, to the best of our knowledge, the first report describing the formation of amino acid conjugates for this class of compounds. This finding uncovers a previously unknown metabolic pathway for iridoids, thereby expanding current understanding of their biotransformation and opening new perspectives on their metabolic fate and potential biological implications. The detection of putative amino acid conjugates, although not confirmed with authentic standards, may be mechanistically supported by recent evidence indicating that iridoid scaffolds are capable of forming conjugates with peptides in synthetic systems [39]. In this respect, future studies could test the validity of this proposed pathway by systematically evaluating the reactivity of iridoid intermediates with a range of biologically relevant amino acids under physiologically relevant conditions [39]. In biological systems, such conjugation reactions are typically preceded by phase I transformations that generate more reactive intermediates, which can subsequently undergo conjugation to increase polarity and facilitate excretion [19].

### 3.6. Predictive Capacity and Limitations of in Silico Approaches

In the context of the present study, it is important to consider that both the in silico predictions and the in vitro microsomal assays represent simplified experimental approaches that do not fully replicate whole-organism metabolism. While in silico models provide insights into potential biotransformation pathways based on structural rules, they do not account for physiological factors such as tissue-specific enzyme expression, transporter activity, or systemic interactions [40]. Similarly, liver microsomes primarily reflect hepatic enzymatic activity (mainly phase I oxidation and selected phase II conjugation) while lacking key processes including absorption, distribution, extrahepatic metabolism, and elimination. Consequently, the metabolic profiles obtained in this study can be interpreted as indicative of potential pathways rather than definitive representations of in vivo biotransformation. In this context, the present results should be considered as hypothesis-generating and useful for prioritizing metabolites, with an aim of guiding future in vivo studies and confirmatory analyses, in line with current Next Generation Risk Assessment (NGRA) approaches [41].

Moreover, in the present study, a discrepancy was observed between the in silico predictions and the experimentally identified metabolites. Recent reviews have highlighted the underlying mechanisms that may explain discrepancies between in silico predictions and in vitro metabolic pathway results. These differences are largely attributed to the inherent simplifications of computational models, limitations in experimental systems such as microsomal assays, incomplete representation of enzymatic pathways (particularly for phase II metabolism), and variability in enzyme expression, transport processes, and species-specific factors [40]. On the one hand, the ADMETlab model successfully predicted the rapid metabolic clearance of NL, which is consistent with the fast biotransformation observed in the in vitro microsomal assays. This prediction is likely associated with the high probability of NL being a substrate of CYP3A4, a major enzyme involved in xenobiotic metabolism. On the other hand, BioTransformer 4.0 showed limited accuracy in predicting the specific metabolites detected experimentally [42]. This divergence may be attributed to the inherent limitations of rule-based in silico tools [40], which may not fully capture complex, multi-step metabolic pathways, enzyme specificity, or less common reactions such as those observed in this study. In particular, the requirement of sequential transformations and the formation of intermediate metabolites may not be adequately represented in current prediction models [38].

## 4. Conclusions

This study aimed to provide the first integrated toxicokinetic characterization of pure NL by combining in silico ADMET predictions with in vitro rat and human liver microsomal metabolism and UHPLC–MS/MS–based metabolite identification. NL was shown to undergo rapid and extensive hepatic biotransformation through both phase I oxidative (86% reduction in NL concentration after 120 min) and phase II conjugative (89% reduction in NL concentration after 120 min) pathways, with comparable depletion kinetics in rat and human microsomes, supporting its low metabolic persistence. While phase I metabolic routes were largely conserved between species, phase II metabolism was markedly more diverse in human microsomes, revealing relevant interspecies differences in conjugative capacity. Importantly, although oxidoreduction and hydrolysis reactions were previously reported for other iridoids, this study suggests the possible formation of previously unreported amino acid-related derivatives, although these require further confirmation. Nevertheless, certain limitations should be acknowledged. The in vitro microsomal model does not fully replicate the complexity of whole-organism metabolism, including extrahepatic pathways, transporter involvement, and systemic distribution processes. Overall, these findings significantly expand current understanding of NL and iridoid biotransformation and provide mechanistically relevant data to support future in vivo studies, interspecies extrapolation, and human and environmental risk assessment of NL-containing products.

## Figures and Tables

**Table 1:** Validation parameters, including recovery (%), within-day repeatability (Sw); between-day repeatability (SB); intermediate precision (S_IP_); relative standard deviation (%RSD_IP_); and limits of detection (LOD) and quantification (LOQ) for Nepetalactone, at three concentration levels, in three different days. RSD_AOAC_ (%): ≤11–16% for 100 and 500 µg L^−1^, and ≤8% for 1000 µg L^−1^. Acceptable Recovery Range (%) by AOAC: 80–110% for 100, 500 and 1000 ppb.

NL Concentration (µg L^−1^)	Recovery (%)	S_W_	S_B_	S_IP_	RSD_IP_ (%)	LOD (µg L^−1^)	LOQ (µg L^−1^)
100	101.64	5.68	17.52	11.13	10.95	0.018	0.059
500	103.20	6.36	67.50	39.32	7.62		
1000	103.99	14.54	91.24	54.00	5.19		

**Table 2:** Tentative identification of potential phase I and phase II metabolites following in vitro Nepetalactone metabolism with female (FRLM) rat and human (HLM) microsomes by UHPLC-MS/MS.

Formula	ID	Transformations	Composition Change	*m*/*z* Error (ppm)	*m*/*z* (obs.)	*m*/*z* (calc.)	RT (min)	Area (Max.)	FRLM	HLM
**Phase I**										
C_10_H_12_O_2_	M22	Desaturation	−(H_2_)	−1.5	165.09076	164.08348	4.2	4,692,581	+	+
C_10_H_16_O_3_	M23	Hydration	+(H_2_O)	−1.62	185.11692	184.10965	4.0	5,782,264	+	+
C_10_H_16_O_2_	M24	Reduction	+(H_2_)	−2.18	169.12194	168.11466	3.7	4,562,605	−	+
**Phase II**										
C_13_H_19_NO_3_S	M25	Dehydration, Cysteine Conjugation *	+(C_3_H_5_NOS)	−1.11	270.11554	269.10826	4.4	5,920,562	−	+
C_13_H_21_NO_4_S	M26	Cysteine Conjugation *	+(C_3_H_7_NO_2_S)	−1.47	288.12598	287.11871	3.3	5,912,325	−	+
C_12_H_21_NO_2_	M27	Hydration, Nitro Reduction, Glycine Conjugation *	+(C_2_ H_7_ N)	−1.9	212.1641	211.15683	3.8	3,081,609	−	+
	M28	Nitro Reduction, Glycine Conjugation *							−	+
C_12_H_20_O	M29	Nitro Reduction, Reduction, Acetylation	−(O) + (C_2_H_6_)	−1.53	181.15842	180.15114	6.7	2,041,546	+	-
C_28_H_46_O_3_	M30	Desaturation, Stearyl Conjugation	+(C_18_H_32_O)	−1.09	431.3515	430.34423	7.3	7,676,211	−	+
C_11_H_18_	M31	Reduction, Methylation	−(O_2_) + (CH_4_)	−0.96	151.14798	150.14071	5.1	4,955,747	−	+
C_11_H_16_	M32	Desaturation, Reduction, Methylation	−(O_2_) + (CH_2_)	−0.51	149.1324	148.12513	4.2	3,871,240	−	+
C_13_H_19_NO_3_S	M33	Dehydration, Cysteine Conjugation *	+(C_3_H_5_NOS)	−0.55	270.11569	269.10842	4.4	2,362,976	−	+
C_16_H_28_N_4_O_2_	M34	Nitro Reduction, Oxidation, Arginine Conjugation	+(C_6_H_14_N_4_)	−4.51	309.22711	308.21984	3.6	2,002,612	−	+
	M35	Nitro Reduction, Arginine Conjugation							−	+

* Tentative cysteine/glycine-related adducts.

## Data Availability

The data presented in this study are available on request from the corresponding author.
